# Study of a BALB/c Mouse Model for Allergic Asthma

**DOI:** 10.5487/TR.2008.24.4.253

**Published:** 2008-12-01

**Authors:** Young-Su Yang, Mi-Jin Yang, Kyu-Hyuk Cho, Kyuhong Lee, Yong-Bum Kim, Jin-Sung Kim, Myung-Gyun Kang, Chang-Woo Song, Chang-Woo Song

**Affiliations:** 13 Division of Inhalation Toxicology, Korea Institute of Toxicology, Korea Research Institute of Chemical Technology, 1051 Shinjeong-dong, Jeongeup, Jeollabuk-do, 580-185 Republic of Korea; 23 Division of Toxicological Pathology, Korea institute of Toxicology, Daejeon, 305-343 Korea; 33 Division of Toxicology, Korea institute of Toxicology, Daejeon, 305-343 Korea

**Keywords:** Asthma, Mouse, Eosinophil, Immunoglobulin E, Cytokine, Model

## Abstract

Allergic asthma is a worldwide public health problem and a major socioeconomic burden disease. It is a chronic inflammatory disease marked by airway eosinophilia and goblet cell hyperplasia with mucus hypersecretion. Mouse models have proven as a valuable tool for studying human asthma. In the present report we describe a comparison of mouse asthma models. The experiments were designed as follows: Group I was injected with ovalbumin (OVA, i.p.) on day 1 and challenged with 1% OVA (aerosol exposure) on days 14~21. Group II was injected on day 1, 14 and aerosol-immunized on days 14~21. Group III was injected on day 1, 14 and immunized by 1% OVA aerosol on days 18~21. We assessed asthma induction by determining the total number of white blood cells (WBC) and eosinophils as well as by measuring cytokine levels in bronchoalveolar lavage fluid (BALF). In addition, we evaluated the histopathological changes of the lungs and determined the concentration of immunoglobulin E (IgE) in serum. Total WBC, eosinophils, Th2 cytokines (IL-4, IL-13) and IgE were significantly increased in group I relative to the other groups. Moreover, histopathological studies show that group I mice show an increase in the infiltration of inflammatory cell-in peribronchial and perivascular areas as well as an overall increase in the number of mucus-containing goblet cells relative to other groups. These data suggest that group I can be a useful model for the study of human asthma pathobiology and the evaluation of existing and novel therapeutic agents.

## INTRODUCTION

The severity and mortality of asthma are rapidly elevating around the world. There are some reports that 17 million Americans were affected by bronchial asthma in 1996, and the economic burden is increasing in parallel (US $ 6.2 billion in 1990, US $ 12.7 billon in 1998) (Weiss and Sullivan, 2001). In Asia, specifically developed countries, asthma patients are increasing more rapidly. As an example, Chinese asthmatic patients are estimated to number around 150 million individuals by the year 2013 (Armin Braun *et al.*, 2007). Of even more concern is that the prevalence in children has increased (Paramesh, 2002; Yuk *et al.*, 2007).

In humans, asthma is an allergic disorder which is an IgE-dependent, type 1 hypersensitivity reaction. Clinical symptoms include episodic cough, wheezing, breathlessness, chest tightness and variability in lung function (Meryl *et al.*, 2001; Tattersfield *et al.*, 2002). It is characterized by airway hyperresponsiveness (AHR) and chronic inflammation of the airway walls with infiltration by lymphocytes and eosinophils (Bousquet *et al.*, 1990, 2000). In addition, airway morphology in asthma displays not only the characteristics of an acute inflammatory process but also structural changes (McDonald, 2001; Johnson *et al.*, 2001). It is driven by the activation of mast cells, alveolar macrophages, dendritic cells and airway epithelia cells. These cells release prostaglandin, histamine, cytokines and chemotactic factors (Heo and Kim, 2002; Hamid *et al.*, 2003).

Animal models are useful tools allowing for the study of development and progression of diseases in humans, and animal models of asthma have been used for over 100 years (Karol, 1994). Several animal models have been developed for the study of asthma pathogenesis.

**Table 1.** Representation of protocols for asthma induction in mice

| Strain | Ages & Gender | Sensitization | Exposure | Author |
|---|---|---|---|---|
| BALB/c | 7 weeks, male | 100 μg OVA + 1.6 mg Alum (i.p.) Day 1 | 20 μg OVA/ 50 μl PBS from day 11 to 14 (IN) | El-Hashim *et al.* (2002) |
| BALB/c, C57BL/6, C3H/He, A/J | 5 weeks, male | 10 μg OVA + Alum gel (i.p.) Day 1 and 14 | 1 mg/ml OVA 50 μl from day 21 to 25 (IN) | Kazuhiko Shinagawa and Masami Kojima (2003) |
| BALB/c | 6 weeks, female | 20 μg OVA + 1 mg alum, day 1 and 10 (i.p.) | OVA (10 mg/ml) day 17, 24 and 31 (Aero) | Jiang *et al.* (2005) |
| BALB/c | 25~31 g, male | 20 μg OVA + 1 mg alum, day 1 (i.p.) | 5% OVA from day 14 to 21 (Aero) | Park *et al.* (2007) |
| BALB/c | 5-7weeks, male | 20 μg OVA + 4 mg alum, day 1 and 14 (i.p.) | 1% OVA from day 18 to 20 (Aero) | Fred Wong *et al.* (2007) |
| BALB/c | 6~8 weeks, female | 20 μg OVA + 1 mg Alum (i.p.) Day 1 and 14 | 5% OVA 1 h/day from day 21 to 27 (Aero) | Yuk *et al.* (2007) |
| BALB/c | 20~25 g, male | 100 μg OVA + 10%, 50 mg Alum (i.p.) Day 1 and 6 | 2 % OVA during 6 weeks 3 days/week (Aero) | Sofia *et al.* (2008) |
| C57BL/6J | 17~24 g, female | 25 μg OVA + 2 mg alum, three weekly (i.p.) | 7 days later 1% OVA (Aero) for 3 days (nose-only exposure chamber) | Eric *et al.* (2008) |

OVA: ovalbumin, Alum: aluminum hydroxide, IP: intra-peritoneal, IN: intra-nasal using micropipette, Aero: Aerolized using ultrasonic nebulizer

Cats may develop a bronchial disease that is similar to human chronic asthma (Padrid, 1992), and both sheep (Abraham, 1995) and dogs (De Weck *et al.*, 1997) are known to have a natural susceptibility to some allergens. Guinea-pig models of asthma can provide the essential hallmarks of asthma, including dual bronchoconstrictor responses (Itoh *et al.*, 1996; Toward *et al.*, 2004). However, asthma models in mice are potentially more useful due to the fact that: i) their immune system has been widely characterized, ii) genetically modified mice are available and iii) a wide range of species-specific reagents can be obtained (Hessel *et al.*, 1995; Torres *et al.*, 2005). There have been a number of studies using acute exposures of mice to allergen, as shown in Table 1 (El-Hashim *et al.*, 2002; Jing *et al.*, 2005; Eric *et al.*, 2008).

In the present work, we examined which mouse models are effective for investigation of human asthma. Asthma mice groups were designed based on preliminary studies in our laboratory and other studies in Table 1. Various parameters, such as differential cell count in a BALF (bronchoalveolar lavage fluid), histological examination and cytokines levels were measured as an indicator of asthma. These parameters are useful for determining asthma induction (El-Hashim *et al.*, 2002; Jiang *et al.*, 2005; Eric *et al.*, 2008), and they play an important role in human asthma (Yoshida *et al.*, 2005).

## MATERIALS AND METHODS

***Chemicals.*** Chemicals were purchased from the following sources: Ovalbumin (OVA) from Sigma Co.; aluminum hydroxide from Pierce biotechnology, Inc.; Interleukin-4 (IL-4), Interleukin-5 (IL-5), Interleukin-13 (IL-13) and Immunoglobulin-E (IgE) immunoassay kits from Biosource international, Inc.; other chemicals were of the highest commercial grade available.

***Animals and treatment.*** Female BALB/c mice (6 weeks old, Oriental Co., Ltd. Kyounggi, Korea) were kept in a storage room under the following conditions during the experiments: constant temperature (23±3°C), relative humidity (50±10%), and illumination (12 h light/dark cycles). All animals were fed with standard animal chow daily and had accessed to drinking water *ad libitum*. The mice were divided into the following four groups: (control group, asthma induction group I, II and III), and each group consisted of ten animals. For sensitization, group I, II, and III were treated with 20 μl of ovalbumin with 1 mg of aluminum hydroxide in 500 μl of saline, whereas the control group received 500 μl of saline intraperitoneally on day 1. Group II and III were treated for the second sensitization on day 14. As shown in Fig. 1, group I and II were challenged once daily from day 14 to day 21 by exposure to an aerosol of 1% OVA in saline using an ultrasonic nebulizer (3 ml/min, Omron, Tokyo, Japan). Group III was challenged three times a day from day 18 to day 21 by exposure to an aerosol of 1% OVA in saline. Mice of the control group were challenged with nebulized saline. All the animal facilities in this study were accredited by the Association for Assessment and Accreditation of Laboratory Animal Care International (AAALAC).

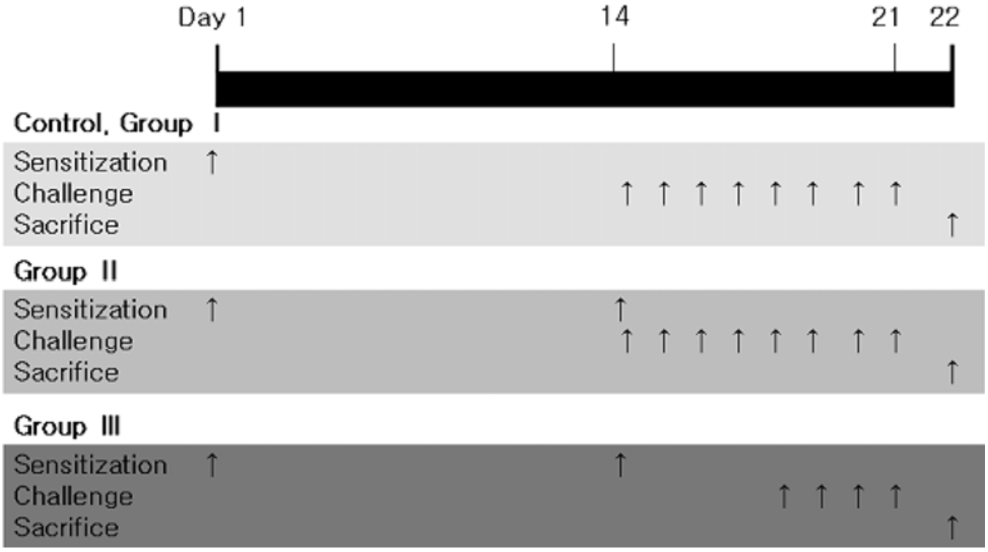

**Fig. 1.** Schematic diagram of an experimental protocol. The mice were divided into four groups, and asthma induction was in three groups. The control group was sensitized and challenged with 0.9% saline. The group I was immunized through intraperitoneal (i.p.) injection with 20 μg of chicken OVA and 1 mg of aluminum hydroxide on day 1 but, the group II and III on days 1 and 14. The mice were exposed to a 1% ovalbumin solution aerosolized using and ultrasonic nebulizer for 30 min per one time on day from days 14 to 21, but the group III exposed for 30 min per three times on day from days 18 to 21.

***Collection of BALF (bronchoalveolar lavage fluid) and blood.*** Using isoflurane, the sensitized mice and the control mice were anesthetized, respectively. Blood was collected from the caudal vena cava for serum IgE measurements. The trachea of anesthetized mice was cannulated. Next, both lungs of each mouse tagged with an even number was lavaged with 1 ml of PBS three times. The collected BALF was pooled, and centrifuged at 3000 rpm for 4 min. The pellet was re-suspended with 500 μl of PBS.

***Histological examination.*** The lung tissue of each mouse tagged with an odd number was fixed in a 10% formaldehyde solution at room temperature for 2 days, and then was paraffin-embedded. The tissues were sectioned at a thickness of 4 μm for histology. The sections were de-paraffinized with xylene, and then stained with haematoxylin and eosin (H&E) and periodic acid-Schiff (PAS) stain. All sections of the stained tissue were analyzed by bright-field microscopy.

***Measurement of BALF cytokines and differential cell counts.*** Cytokine levels in BALF supernatant were measured using ELISA kits (Biosource, USA) according to the manufacturer's instruction. IgE from serum was calculated by same method. Total and differential cell counts were performed using an ADVIA120 Hematology system (Bayer, USA).

***Statistical analysis.*** Statistical analyses were performed using Statistical Analysis Systems (SAS/STAT User's Guide Version 8.2, NC, USA). For all parameters, Bartlett's test was performed according to whether a significant interaction was present or not, Dunn's Rank Sum and ANOVA tests were used to compare the control group with experimental groups. Only if the differences exist. Student's t-test was performed to test differences between a pair of each group. All results were calculated as the means ± standard error of the mean and differences were considered significant when $p < 0.05$ or $p < 0.01$.

## RESULTS

We induced asthma in BALB/c mice using the protocol illustrated in Fig. 1, and assessed the severity of asthma induction by counting the total number of white blood cells, eosinophils and by measuring cytokine levels in BALF. Also, the histopathological changes in lung tissue were characterized and the levels of immunoglobulin E (IgE) in serum were estimated.

***Total leukocytes and eosinophils in BALF.*** Mice immunized with OVA/alum and then challenged with OVA aerosol showed significant increases in the total white blood cells in BALF (Fig. 2A). The percentage of eosinophils was 4.06 ± 1.51% in control mice, 46.54 ± 6.88% in group I mice, 38.20 ± 4.80% in group II mice,

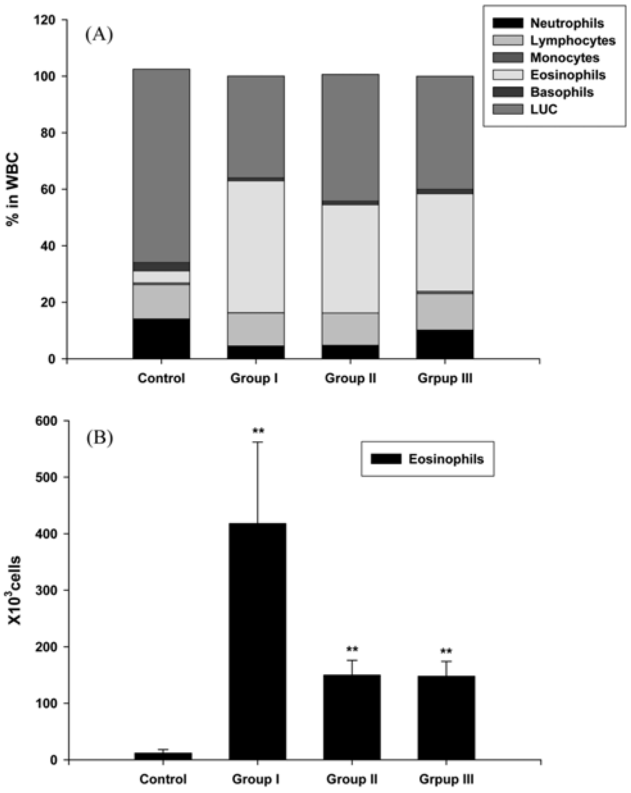

**Fig. 2.** Total leukocytes and eosinophils in BALF. Mice were killed 24 h after the last OVA challenge. The bronchoalveolar lavage fluid (BALF) was collected and analyzed using an ADVIA 120. (A) The percentage of each cell type in white blood cells (WBC) is shown. LUC; large unstained cells. (B) Total numbers of eosinophils in BALF. Values are means ± SEM, n = 10. **p* Value less than 0.05, ***p* value less than 0.01 versus control group.

34.38 ± 3.64% in group III mice, respectively. Correspondingly, the number of eosinophils showed a marked increase (Fig. 2B). The numbers of eosinophils in BALF in group I (418 ± 144), group II (150 ± 26) and group III (148 ± 26) were increased in comparison with the control group (12 ± 6). Interestingly, the levels of eosinophils in group I are nearly three times as high as in the other two experimental groups.

***Th2 cytokine levels in BALF and IgE levels in serum.*** Th2 cytokines play important roles in asthma during airway remodeling and the development of AHR. To determine the levels of cytokines (IL-4, IL-5 and IL-13) in BALF after repeated antigen exposure, ELISA studies were performed. IL-4 levels in BALF were slightly, but non-significantly, elevated in asthma induction groups, with the largest increase seen in group I (Fig. 3A). Levels of IL-5 and IL-13 were significantly increased in the asthma induction groups, except for group III in IL-5 level (Fig. 3B and 3C). In particular, IL-13 level in group I was significantly high compared with the level in other asthma induction groups ($p < 0.01$).

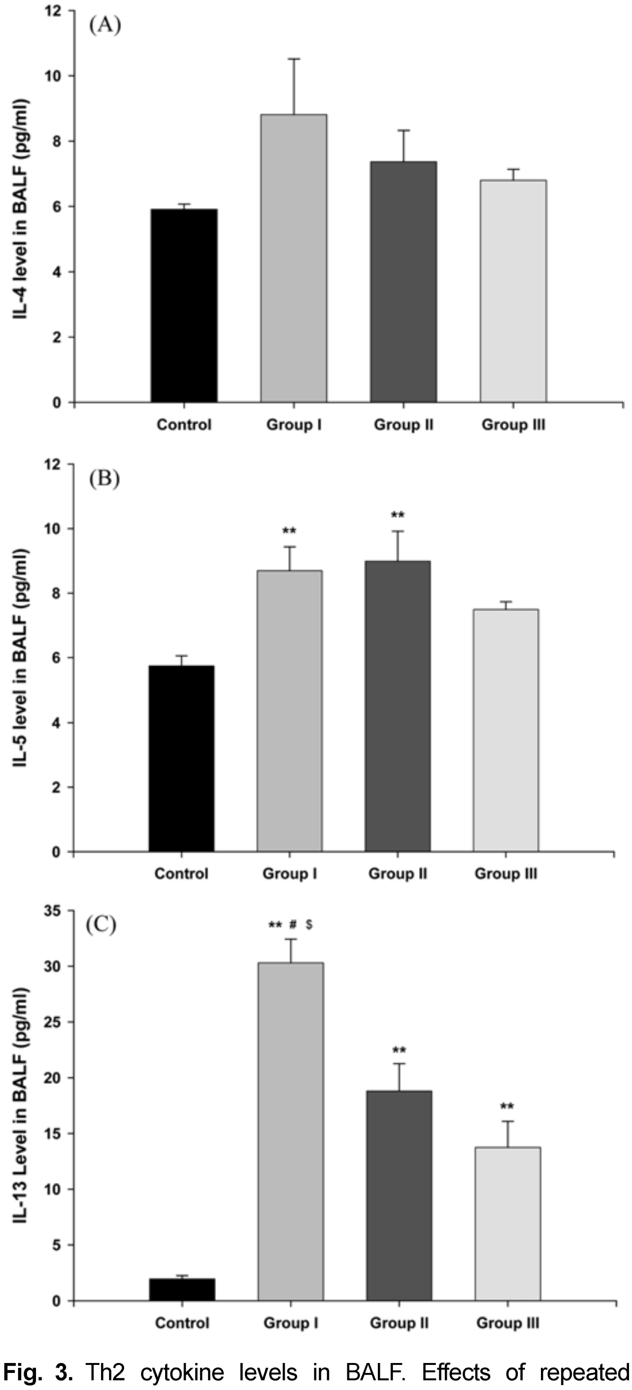

**Fig. 3.** Th2 cytokine levels in BALF. Effects of repeated ovalbumin (OVA) exposure on the IL-4, IL-5 and IL-13 levels in BALF. The IL-4, IL-5 and IL-13 levels in BALF were analyzed by ELISA as described in the Materials and Methods section. Values are means ± SEM, n = 5. ***p* value less than 0.01 versus control group; $^{\#}p < 0.01$ group I vs group II; $^{\$}p < 0.01$ group I vs group III.

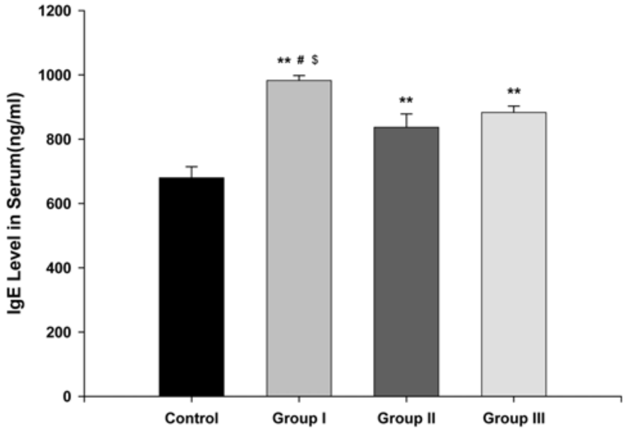

**Fig. 4.** Immunoglobulin-E levels in serum. Effects of repeated ovalbumin (OVA) exposure on the IgE levels in serum. The IgE level in the serum was analyzed by ELISA as described in the Materials and Methods section. Values are means ± SEM, n = 10. $^{**}p$ value less than 0.01 versus control group; $^{\#}p < 0.01$ group I vs group II; $^{\$}p < 0.01$ group I vs group III.

The serum levels of total IgE in all groups were significantly increased when compared with control mice (Fig. 4). Moreover, IgE level in group I showed statistical significancy compared to the level in other asthma induction groups ($p < 0.01$).

***Histopathologic response of lung tissues.*** We next made a histological analysis of lung sections taken from the four groups of mice after OVA exposure by nebulization. The morphological features of lung sections, which were stained with of Haematoxylin and eosin (H&E), are shown in Fig. 5. Asthma induction mice (Group I, II and III) showed a marked increase in the infiltration of inflammatory cells in the perivascular, bronchus and bronchiole areas (Fig. 5B, 5C and 5D, arrow). Many of the epithelial cells seemed to be enlarged due to the accumulation within their cytoplasm of homogeneous-looking material that stained positively with periodic acid-Schiff (PAS). PAS staining of lung tissue demonstrated a marked increase in goblet cells containing mucus as well as cell proliferation in the bronchial epithelium of OVA-allergic mice groups (Fig. 6B, 6C and 6D, arrow), as compared to the control group (Fig. 6A). Importantly, among the OVA-allergic mice groups, the incidence and severity of the

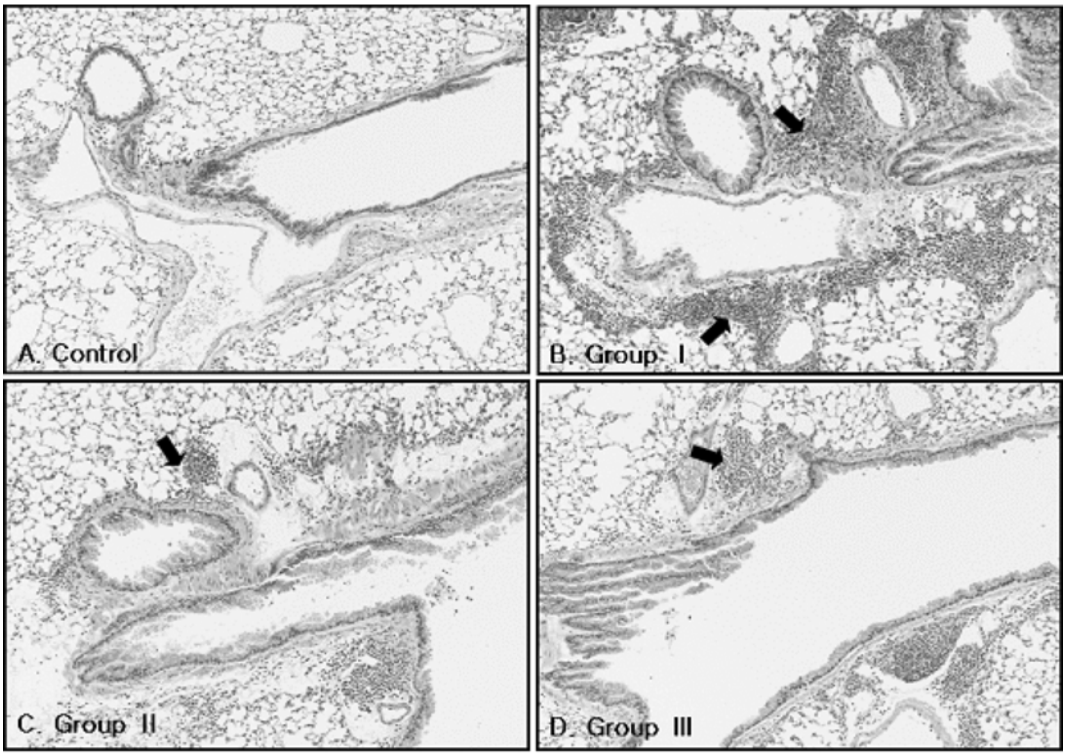

**Fig. 5.** Histopathologic response of lung tissues (H&E). Lung tissues were excised from control group or ovalbumin-induced mice group I, group II or group III. The control group was sensitized and challenged with 0.9% saline. Group I, II and III were sensitized and challenged with OVA and alum according to the experimental protocol. Tissue sections were stained with H&E as described in the Materials and Methods section. In asthma group mice, increases in inflammatory cells (arrows) were apparent around the airways (Magnification, × 200).

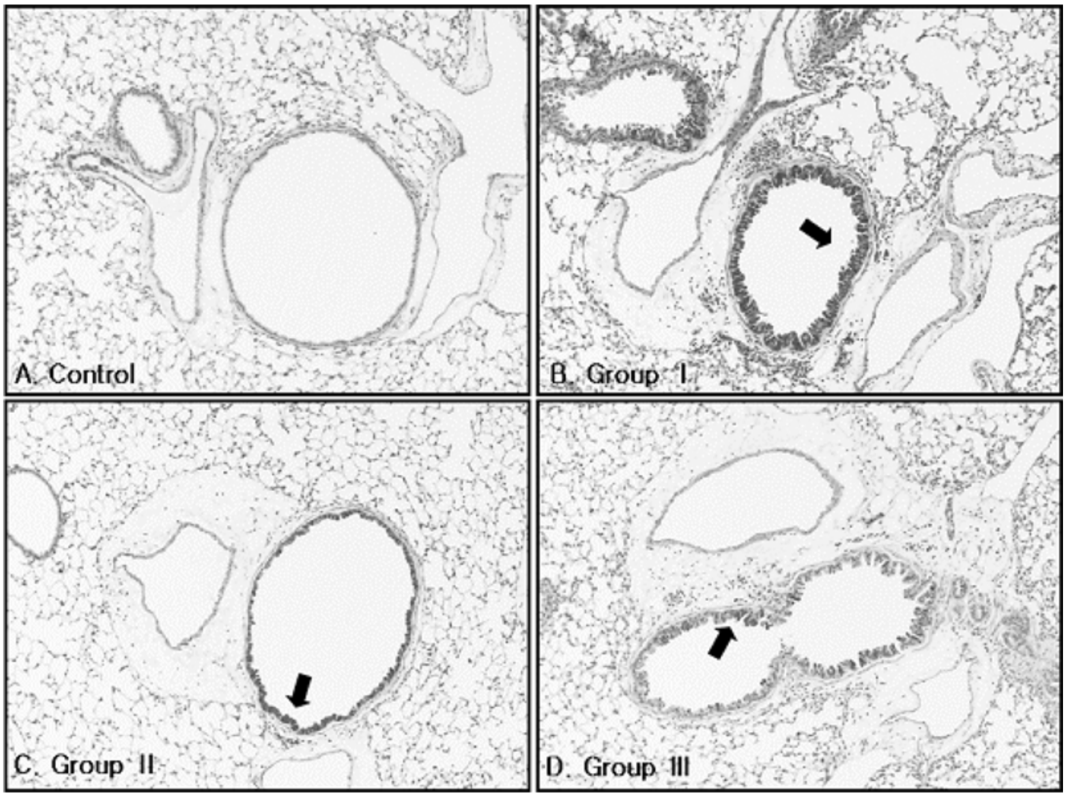

**Fig. 6.** Histopathologic response of lung tissues (PAS). Lung tissues were excised from control group or ovalbumin-induced mice group I, group II or group III. The control group was sensitized and challenged with 0.9% saline. Group I, II and III were sensitized and challenged with OVA and alum according to the experimental protocol. Tissue sections were stained with periodic acid-Schiff (PAS) stain as described in the Materials and Methods section. Arrows indicates the mucus hypersecretion (Magnification, × 200).

**Table 2.** Effects of repeated ovalbumin (OVA) exposure on mice lung tissue. Incidence and severity of microscopic findings were described. The grade of inflammation cell infiltration and mucus hyperplasia was assessed in a blinded fashion on a scale of from minimal to marked by examining 10 randomly chosen regions per sample at a magnification of × 200. The extent of lesions was graded as minimal (< 25% of the slide), mild (25~50%), moderate (50~75%), and marked (> 75%)

| Group | | Female | | | |
|---|---|---|---|---|---|
| | | Control | Group I | Group II | Group III |
| Lung | | 10 | 10 | 10 | 10 |
| Inflammatory cell infiltration | Minimal | 0 | 0 | 0 | 1 |
| | Mild | 0 | 3 | 6 | 4 |
| | Moderate | 0 | 6 | 4 | 5 |
| | Marked | 0 | 1 | 0 | 0 |
| Mucus hyperplasia | Minimal | 1 | 0 | 0 | 0 |
| | Mild | 0 | 0 | 2 | 4 |
| | Moderate | 0 | 6 | 8 | 6 |
| | Marked | 0 | 4 | 0 | 0 |

histopathological changes in the lungs were highest in group I (Table 2).

## DISCUSSION

Many kinds of animal models have been developed to study the pathobiology of asthma; however, mice model are more useful than the others because: i) their immune systems are largely characterized, ii) genetically modified animals are available and iii) a wide range of species-specific reagents can be obtained (Torres *et al.*, 2005; Hessel *et al.*, 1995). As shown in Table 1 there are many variations on asthma mouse models, each with different protocols for sensitization and exposure periods. In this study, we have selected the BALB/c strain, because it produce higher levels of anti-ovalbumin IgE antibody and Th2 cytokines than other strains (Rakesh *et al.*, 2008). For sensitization, ovalbumin and aluminum hydroxide are most commonly used with various doses for intraperitoneal injection as shown in Table 1 (Fred Wong *et al.*, 2007; Park

*et al.*, 2007; Yuk *et al.*, 2007; Rakesh *et al.*, 2008).

The aim of this study is to establish the most efficient mouse model for investigation of human asthma. Asthma mice groups were designed based on preliminary studies in our laboratory and other studies in Table 1. Therefore, we chose to sensitize with 20 μg ovalbumin + 1 mg aluminum hydroxide for 7 weeks old mice. There are some reports about the impact of the size and type of exposure chamber on the results of similar experiments; for example, Sofia *et al.* (2008) used a chamber measuring 14. 5 cm × 28.5 cm × 15 cm, and Jiang *et al.* (2005) used a chamber measuring, 440 $cm^3$ (diameter 12 × 12 cm, height 10 cm). Interestingly, a tetrahedron-shaped chamber was not good for asthma induction because mice were crowed in a corner of the chamber. Consequently, some mice could not be induced properly leading to large deviation of parameter's value. Therefore, in order to remove this problem, a cylindrical exposure chamber was used in this study (radius 12.5 cm, height 10 cm).

Based on the above information, three groups of allergic asthma BALB/c mice models were designed, group I and group II were planned to determine the sensitization effect while group III was intended to allow for examination of exposure time effect. The results of these studies indicate that all three of these asthma induction models (especially group I) were useful for evaluating asthma, as demonstrated by the following four main reasons;

(1) Eosinophilic inflammation was observed in the asthma induction groups. Mice immunized with OVA/alum and then challenged with an OVA aerosol showed a significant increase in total white blood cell count in BALF when compared with the control group. Specifically, the numbers of eosinophils in BALF of group I, II and III were significantly higher than the control group. Eosinophils are implicated as key effector cells in asthmatic airways since they secrete cytotoxic proteins and lipid mediators that have the capacity to promote pathological changes believed to contribute to the decline in lung function (Martin *et al.*, 1996).

(2) Th2 Cytokine levels in BALF were elevated in the asthma induction groups, relative to the control group. In humans, expression levels of IL-4, IL-5 and IL-13 were increased in the bronchial mucosa of human asthmatics (Kay, 1997; Humbert, 1997). Studies concerning asthma suggest the importance of Th2 cytokines secreted by resident cells, such as epithelial cells, macrophages and mast cells, as well as infiltrated cells (eosinophils and lymphocytes) (Yuk *et al.*, 2007; Barnes *et al.*, 1998). IL-4 and IL-13 play important roles in IgE switching in B cells, the development of eosinophil infiltration into the airways and mucus hypersecretion (Kips, 2001). IL-5 is an essential molecule for the terminal differentiation, migration, and prolonged survival of eosinophils and airway hyper-responsiveness (Cho *et al.*, 2004; Kips, 2001).

(3) IgE levels in serum were elevated in the asthma-induced groups, as shown Fig. 4. The asthmatic response after antigen inhalation in patients with allergic asthma results from an IgE-dependent type-1 hypersensitivity reaction (Hamid *et al.*, 2003). IgE is associated with the early phase of allergic asthma (Kim and Heo, 2001). After the binding of the allergen to IgE, the complex interacts with the IgE receptor and activates mast cells to secret numerous mediators, causing symptoms of the disease to worsen (Owen, 2007).

(4) Marked histological changes were observed in the asthma models. In our histological investigation, inflammatory cells had infiltrated into the perivascular, bronchus and bronchiole area. Moreover, PAS staining of lung tissue demonstrated a marked increase in the number of goblet cells containing mucus as well as an increase in cell proliferation in the bronchial epithelium of OVA-allergic mice groups, when compared with the control group.

As shown above, all asthma-induced models described in this study were successful in the expression of asthma symptoms, especially group I. It can not be clearly determined why this is the case, however, we speculate that it may be a result of antigen-tolerance. Swirls and coworkers recently reported the importance of the granulocyte-macrophage colony-stimulating factor in BALB/c mice which are chronically exposed to antigen (Swirls *et al.*, 2002). They showed that in antigen-tolerant mice, eosinophilia was fully restored by repeated antigen exposure. Another study showed that no airway lesions were seen and that no AHR was elicited in C57BL/6 mice when they were exposed to low levels of aerosolized antigen (Kazuhiko Shinagawa and Masami Kojima, 2003; Temelkovski *et al.*, 1998; Kumar and Foster, 2002). In addition, some hypotheses can be made based on this: (1) The Exposure period was more important than number of sensitization in regulating the number of eosinophils. As shown in Fig. 2(B) & Fig. 3(C), the number of eosinophils and the concentration of IL-13 in group I were higher than those of other asthma groups, while IL-13 values in group II and III were similar. (2) IL-5 and IgE levels are not affected by exposure periods or the number of sensitizations. Since all asthma-induced groups showed similar levels of both. However, a more detailed examination is needed to confirm these results and to better understand the mechanisms of asthma. It was clear that the protocol used for antigen sensitization/challenge is very important to sustaining the allergic reaction.

In summary, to make a good asthma model, it appears as though one sensitization is better than more than one and longer challenge periods are better than extensive short periods. If sensitization is to be done twice or for long exposure times, you must increase the OVA concentration during the challenge phase. Asthma models of BALB/c were useful for evaluating asthma, especially group I. We make this claim based on the observed increase seen in: i) total WBC, ii) eosinophils, iii) Th2 cytokines in BALF and iv) IgE levels in serum in addition to the drastic histological changes seen in the lung sections. We speculate that this model will be useful for better understanding human asthma pathobiology and to test potential drug therapies.

## ACKNOWLEDGEMENTS

This study was supported by the Ministry of Science and Technology for the General Project grant of the establishment of the International Inhalation Toxicology Evaluation Technology of Korea Institute of Toxicology.

## References

[CR1] Abraham WM (1995). Animal models of late bronchial responses. Eur. Respir. Rev..

[CR2] Armin B, Thomas T, David AG (2008). Editorial: Experimental models of asthma. J. Occu. Med. and Toxicol..

[CR3] Barnes PJ, Chung KF, Page CP (1998). Inflammatory mediators of asthma: an update. Pharmacol. Rev..

[CR4] Blanchard C, Mishra A, Saito-Akei H, Monk P, Anderson I, Rothenberg ME (2005). Inhibition of human inter-leukin-13-induced respiratory and esophageal inflammation by anti-human interleukin-13 antibody (CAT-354). Clin. Exp. Allergy.

[CR5] Bousquet J, Chanez P, Lacoste JY, Barneron G, Gga-vanian N, Enander I, Venge P, Ahlstedt S, Simony-Lafontaine J, Goard P (1990). Eosinophilic inflammation in asthma. N. Engl. J. Med..

[CR6] Bousquet J, Jeffery PK, Busse WW, Johnson M, Vignola AM (2000). Asthma: from bronchoconstriction to airways inflammation and remodeling. Am. J. Respir. Cht. Care. Med..

[CR7] Cho JY, Miller M, Baek KJ Han JW, Nayar J, Lee SY, McElwain K, McElwain S, Friedman S, Broide DH (2004). Inhibition of airway remodeling in IL-5 deficient mice. J. Clin. Invest.

[CR8] De Weck AL, Mayer P, Stumper B, Schiessel B, Pickat L (1997). Dog allergy, a model for allergy genetics. Int. Arch. Allergy Immunol..

[CR9] El-Hashim AZ, Wyss D, Zuany-Amorim C (2002). Kinetics of airway hyperresponsiveness and airway eosinophilia in BALB/c mice and their modulation by different dexamethasone treatment regimens. Pulmonary Pharmacol. & Thera..

[CR10] Eric R, Secor J, William E, Carson IV, Anurag S, Mellisa P, Linda A, Guernsey CM, Schramm, Roger ST (2008). Oral bromelain attenuates inflammation in an oval-bumin-induced murine model of asthma. eCAM..

[CR11] Flood-Page P, Menzies-Gow A, Phipps S, Ying S, Wangoo A, Ludwig MS, Barnes N, Robinson D, Kay AB (2003). Anti-IL-5 treatment reduces deposition of ECM proteins in the bronchial subepithelial basement membrane of mild atopic asthmatics. J. Clin. Invest..

[CR12] Fred Wonga WS, Hua Z, Wupeng L (2007). Cys-teinyl leukotriene receptor antagonist MK-571 alters bron-choalveolar lavage fluid proteome in a mouse asthma model. Euro. J. Phar.

[CR13] Fulkerson P, Fischetti C, Hassman L, Nikolaidis NM, Rothenberg ME (2006). Persistent effects induced by IL-13 in the lung. Am. J. Respir. Cell. Mol. Biol..

[CR14] Hamid Q, Tulic MK, Liu MC, Moqbel R (2003). Inflammatory cells in asthma: mechanisms and implications for therapy. J. Allergy Clin. Immunol..

[CR15] Heo Y, Kim KH (2002). Development of subacute animal model to predict occurance of systemic anaphylaxis following vaccination and evaluation of various immuno-toxicological parameters. J. Toxicol. Pub. Health.

[CR16] Hessel EM, Van Oosterhout AJ, Hofstra CL D, Bie JJ, Garssen J V, Loveren H (1995). Bronchoconstriction and airway hyperresponsiveness after ovalbumin inhalation in sensitized mice. Eur. J. Pharmacol..

[CR17] Humbert M, Durham SR, Kimmitt P, Powell N, Assoufi B, Pfister R, Menz G, Kay AB, Corrigan CJ (1997). Elevated expression of messenger ribonucleic acid encoding IL-13 in the bronchial mucosa of atopic and nonatopic subjects with asthma. J. Allergy Clin. Immunol..

[CR18] Kim HA, Heo Y (2001). Significance of a highly specific and sensitive enzyme linked immunosorent assay on evaluation of environmental toxicant-mediated allergic responses. J. Toxicol. Pub. Health.

[CR19] Itoh K, Takahashi E, Mukaiyama O, Sathoh Y, Yamaguchi T (1996). Relationship between airway eosi-nophilia and airway hyperresponsiveness in a late asthmatic model of guinea pigs. Int. Arch. Allergy Immunol..

[CR20] Jiang JH, Li G Z, Chai OH, Song CH (2005). Increased intraepithelial mast cells in pulmonary airways of mouse asthma model. The Korean J. Anat.

[CR21] Johnson PRA, Roth M, Tamm M, Hughes M, Ge Q, King G, Burgess JK, Black JL (2001). Airway smooth muscle cell proliferation is increased in asthma. Am. J. Respir. Crit. Care Med..

[CR22] Karol MH (1994). Animal models of occupational asthma. Eur. Respir. J..

[CR23] Kasaian MT, Donaldson DD, Tchistiakova L, Marquette K, Tan XY, Ahmed A, Jacobson BA, Widom A, Cook TA, Xu X, Barry AB, Goldman SJ, Abraham WM (2007). Efficacy of IL-13 neutralization in a sheep model of experimental asthma. Am. J. Respir. Cell. Mol. Biol..

[CR24] Kay AB, Barata L, Meng Q, Durham SR, Ying S (1997). Eosinophils and eosinophil-associated cytokines in allergic inflammation. Int. Arch. Allergy Immunol.

[CR25] Kazuhiko S, Masami K (2003). Mouse Model of Airway Remodeling. Ame. J. Res. Critical Care Med..

[CR26] Kips JC (2001). Cytokines in asthma. Eur. Respir. J. Suppl..

[CR27] Kumar RK, Foster PS (2002). Modeling allergic asthma in mice: pitfalls and opportunities. Am. J. Respir. Cell. Mol. Biol..

[CR28] Martin LB, Kita H, Leiferman KM, Gleich GJ (1996). Eosinophils in allergy: role in disease, degranulation and cytokines. Int. Arch. Allergy Immunol.

[CR29] McDonald DM (2001). Angiogenesis and remodeling of airway vasculature in chronic inflammation. Am. J. Respir. Crit Care Med..

[CR30] Meryl H, Karol JM, Matheson RW, Lange RL, Michael IL (2001). Use of tumor necrosis factor receptor (TNFR)-knockout mice to probe the mechanism of chemically-induced asthma. J. Toxicol. Pub. Health.

[CR31] Owen CE (2007). Immunoglobulin E: role in asthma and allergic disease: lessons from the clinic. Pharmacol. Ther.

[CR32] Padrid P (1992). Chronic lower airway disease in the dog and cat. Probl. Vet. Med..

[CR33] Paramesh H (2002). Epidemiology of asthma in India. Indian J. Pediatr.

[CR34] Park SJ, Shin WH, Seo JW, Kim EJ (2007). Anthocyanins inhibit airway inflammation and hyperre-sponsiveness in a murine asthma model. Food and Chemical Toxicol.

[CR35] Rakesh KK, Cristna H, Paul SF (2008). The “Classical” ovalbumin challenge model of asthma in mice. Current Drug Targets.

[CR36] Sofia FR, William RF, Kenneth JB, Emma JK (2008). Establishing the phenotype in novel acute and chronic murine models of allergic asthma. Internal. Immunopharmcol..

[CR37] Swirski FK, Sajic D, Robbins CS, Gajewska BU, Jordana M, Stampfli MR (2002). Chronic exposure to innocuous antigen in sensitized mice leads to suppressed airway eosinophilia that is reversed by granulocyte macrophage colony-stimulating factor. J. Immunol..

[CR38] Tattersfield AE, Knox AJ, Britton JR, Hall IP (2002). Asthma. Lancet.

[CR39] Temelkovski J, Hogan SP, Shepherd DR, Foster PS, Kumar RK (1998). An improved murine model of asthma: selective airway inflammation, epithelial lesions and increased methacholine responsiveness following chronic exposure to aerosolised allergen. Thorax.

[CR40] Torres R, Picado C, Mora F (2005). Use of the mouse to unravel allergic asthma: a review of the pathogenesis of allergic asthma in mouse models and its similarity to the condition in humans. Arch. Bronconeumol..

[CR41] Toward TJ, Smith N, Broadley KJ (2004). Effect of phosphodiesterase-5 inhibitor, sidenafil (Viagra), in animal models of airways disease. Am. J. Respir. Crit. Care Med..

[CR42] Weiss KB, Sullivan SD (2001). The health economics of asthma and rhinitis. I. Assessing the economic impact. J. Allergy Clin. Immunol.

[CR43] Yang G, Li L, Volk A, Emmell E, Petley T, Giles-Komar J, Rafferty P, Lakshminarayanan M, Griswold DE, Bugelski PJ, Das AM (2005). Therapeutic dosing with anti-interleukin-13 monoclonal antibody inhibits asthma progression in mice. J. Pharmacol. Exp. Ther.

[CR44] Yang G, Volk A, Petley T, Emmell E, Giles-Komar J, Shang X, Li J, Das AM, Shealy D, Griswold DE (2004). Anti-IL-13 monoclonal antibody inhibits airway hyperresponsiveness, inflammation and airway remodeling. Cytokine.

[CR45] Yoshida M, Watson RM, Rerecich T, O’Byrne PM (2005). Different profiles of T-cell IKN-gamma and IL-12 in allergen-induced early and dual responders with asthma. J. Allergy Clin. Immunol..

[CR46] Yuk JE, Woo JS, Yun CY, Lee JS, Kim JH, Song GY, Uang EJ, Hur IK, Kim IS (2007). Effects of lactose-ß-sitosterol and β-sitosterol on ovalbumin-induced lung inflammation in actively sensitized mice. Inter. Immuno..

